# Prevalence of dry eye disease in the elderly: A protocol of systematic review and meta-analysis

**DOI:** 10.1097/MD.0000000000022234

**Published:** 2020-09-11

**Authors:** Xinyue Zhang, Lizhen Wang, Yanlin Zheng, Lu Deng, Xiaoying Huang

**Affiliations:** a Chengdu University of Traditional Chinese Medicine; b Hospital of Chengdu University of Traditional Chinese Medicine, Chengdu, Sichuan, China.

**Keywords:** dry eye disease, elderly, meta analysis and systematic review, prevalence, protocol

## Abstract

**Introduction:**

Dry eye disease (DED) is one of the most prevalent ocular diseases which remains widely underestimated. New lifestyles driven by information technology and the rapid ageing process have brought DED a severe public health concern. And DED is highly related to the reduction in vision-related quality of life and interfere with daily activities. Since advanced age has been suggested as an important risk factor for DED, the aim of our study was to obtain the pooled prevalence of DED in the elderly over 60 years of age.

**Methods and analysis:**

The following databases will be searched from their inception to August 2020: Electronic database includes PubMed, Embase, Cochrane Library, Web of Science, Nature, Science online, Chinese Biomedical Database WangFang, VIP medicine information, and China National Knowledge Infrastructure (CNKI). Primary outcomes: the number of participants and DED cases. Data will be extracted by 2 researchers independently, risk of bias of the meta-analysis will be evaluated based on the Cochrane Handbook for Systematic Reviews of Interventions. All data analysis will be conducted by data statistics software Review Manager V.5.3. and Stata V.12.0.

**Results:**

The results of this study will systematically evaluate the prevalence of DED in the elderly population over 60 years of age.

**Conclusion:**

The systematic review of this study will summarize the current published evidence of epidemiological investigations of DED with advanced age classification.

**Ethics and dissemination:**

This study is a systematic review, the outcomes are based on the published evidence, so examination and agreement by the ethics committee are not required in this study. We intend to publish the study results in a journal or conference presentations.

**Open Science Fra network (OSF) registration number:**

August 12, 2020. osf.io/3jyb4. (https://osf.io/3jyb4).

## 1 Introduction

Dry eye disease (DED) as one of the most prevalent ocular diseases in the world draws a growing public health concern, which remains widely underestimated. It is defined as “a multifactorial disease of the tears and ocular surface that results in symptoms of discomfort, visual disturbance, and tear film instability, with potential damage to the ocular surface. It is accompanied by increased osmolarity of the tear film and inflammation of the ocular surface.”^[1]^ DED was highly correlated with decreased vision related quality of life and disturbance of daily activities. However, due to the diversity of clinical manifestations and diagnostic criteria, and the poor correlation between clinical signs and symptoms,^[2]^ it is difficult to determine its prevalence.

Large epidemiological studies have shown that after the age of 50, the prevalence of DED in women and men increases every 5 years, and the prevalence rate of women is higher than that of men.^[3–9]^ With the aging of the population and the increase of life expectancy, DED will continue to be one of the main reasons for ophthalmic treatment.^[10]^ Therefore, a better understanding of age-related DED and treatment for this particular population is essential. Previous epidemiological evidence has shown that the overall prevalence of DED is between 5% and 87.5% in the general population worldwide.^[9,11–17]^ In mainland China, the prevalence rate of elderly people over 60 is obviously higher, about 34.4%, while the comprehensive prevalence rate is 17%.^[12]^ It is reported that the proportion of regional elderly population in Australia, Taiwan, South Korea and Japan is 57.5%, 33.7%, 33.2%, and 21.6%, respectively.^[9,14,18,19]^ We hypothesized that this wide heterogeneity can be explained by inconsistent diagnostic criteria and multiple risk factors including age, sex, and latitude.

Therefore, our aim is to conduct a systematic review (SR) and meta-analysis to more accurately determine the prevalence of DED in population over 60 years of age, taking into account the diagnostic methods used.

## 2 Methods

### 2.1 Study registration

The protocol of the SR has been registered.

Registration: Open Science Fra network (OSF) registration number: August 12, 2020. osf.io/3jyb4. (https://osf.io/3jyb4). This SR protocol will be conducted and reported strictly according to Preferred Reporting Items for Systematic Reviews and Meta-Analyses (PRISMA) ^[20]^ statement guidelines, and the important protocol amendments will be documented in the full review.

### 2.2 Inclusion and exclusion criteria for study selection

#### 2.2.1 Inclusion criteria

Inclusion criteria are all epidemiological cohort investigations of DED involving over 60 years of age population. All included studies should have a clear assessment and diagnostic method of DED. The language of the trials to be included only Chinese or English.

#### 2.2.2 Exclusion criteria

Following studies will be excluded: Studies that were conducted in subpopulations, such as people with specific diseases (eg, diabetes, hypertension, rheumatoid arthritis, lupus, Sjögren's syndrome, thyroid associated diseases) were excluded.

### 2.3 Outcome measures

The number of participants and DED cases.

### 2.4 Search methods

#### 2.4.1 Search resources

**Figure 1:**
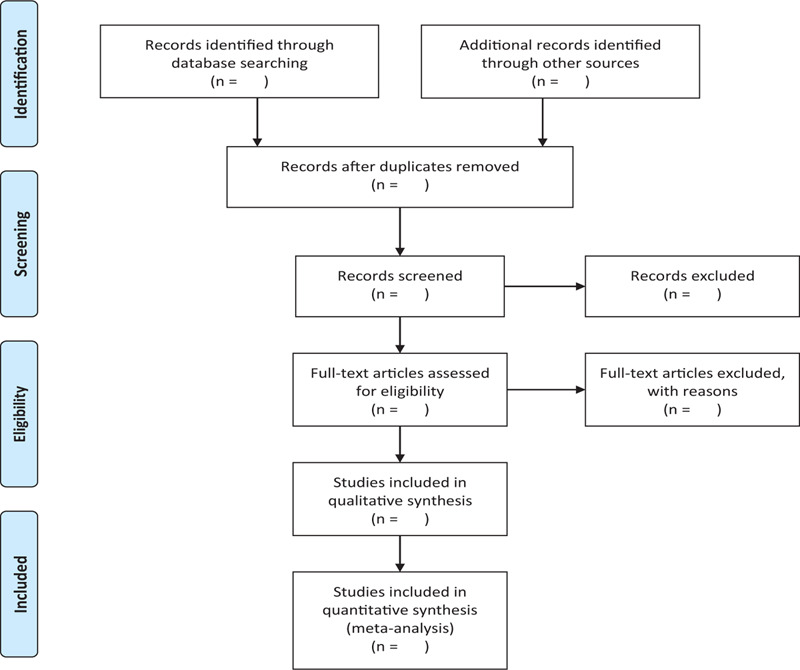
The research flowchart. This figure shows the identification, screening, eligibility, and included when we searching articles.

We will search the following electronic databases from their inception to August 2020: Electronic database includes PubMed, Embase, Cochrane Library, Chinese Biomedical Database, VIP medicine information, and China National Knowledge Infrastructure (CNKI) (Fig. 1 The research flowchart).

#### 2.4.2 Search strategies

The search terms included “prevalence” or “incidence” or “epidemiology” or “morbidity,” combined with “dry eye,” in the forms of free words or controlled vocabulary (ie, medical subject headings). No language restrictions were applied. Second, snowball searching was performed to further identify potentially relevant studies from the reference lists of articles in the first step (Table 1 The research strategy).

**Table 1:**
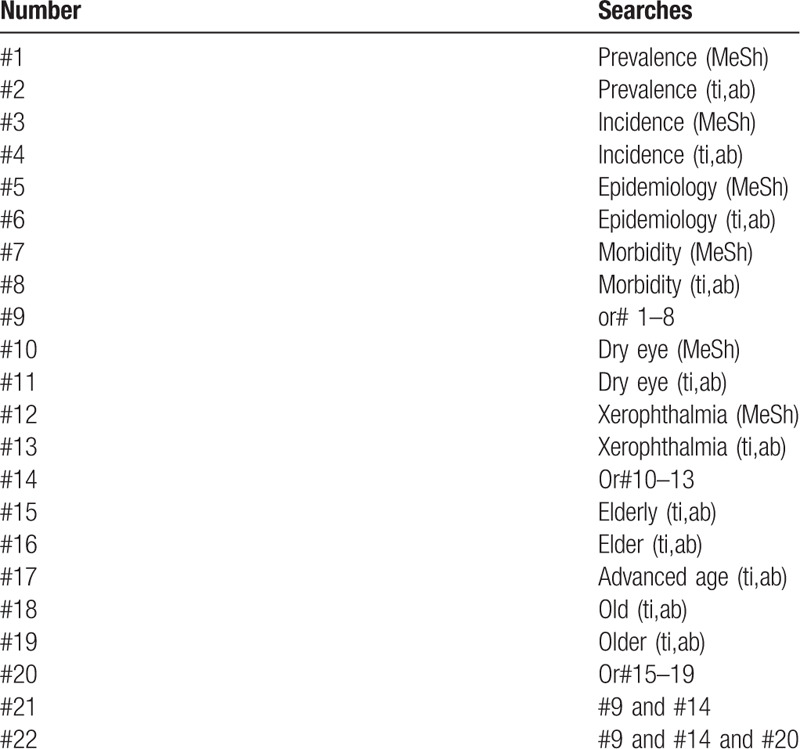
Search strategy sample of PubMed.

### 2.5 Data collection and analysis

#### 2.5.1 Studies selection

There will be 2 researchers (XZ and LW) carry out the selection of research literature independently using Note-Express software. We will first make the preliminary selection by screening titles and abstracts. Secondly, we will download full text of the relevant studies for further selection according to the inclusion criteria. If there is any different opinion, 2 researchers will discuss and reach an agreement. If a consensus could not be reached, there will be a third researcher (YZ) who make the final decision. The details of selection process will be displayed in the PRISMA flow chart.

#### 2.5.2 Data extraction

Two researchers (XZ and LW) will read all the included text in full, and independently extract the following information: The following information will be extracted from each study:

(1) Study characteristics: title, author, study year, study site, diagnostic method.
(2) population characteristics: age, gender.
(3) the outcome measures: the number of participants and DED cases.

We unified the elderly age as over 60. The geographic information (latitude and longitude) of the investigation sites in all included studies was obtained by using the Google Maps GPS coordinates (http://www.gps-coordinates.net/). If the information is not enough, we will contact experts and authors in this field to get relevant information.

#### 2.5.3 Quality of assessment

The STrengthening the Reporting of OBservational studies in Epidemiology (STROBE) criteria were used for checking the quality of reporting. The 21 items identified in the STROBE criteria could achieve a maximum score of 34.

#### 2.5.4 Measures of prevalence

The dichotomous outcomes will be expressed by the odds ratio, while the continuous data will use the standardized mean difference. All these outcomes report 95% confidence intervals.

#### 2.5.5 Assessment of a reporting bias

The bias of publication will be explored through funnel plot analysis. If the funnel plot show asymmetry, it will be evaluated via the Egger and Beg tests, and *P* value <.05 means the publication bias is significant.

#### 2.5.6 Assessment of heterogeneity

The heterogeneity between studies was tested by Cochran *Q* statistic (*Q*-test) and quantified by *I*^2^ index. A *P* < .05 in the *Q*-test indicates significant heterogeneity, and *I*^2^ index represents the percentage of between-study variance due to heterogeneity rather than chance, where values of 25% or less correspond to low heterogeneity, near 50% indicate moderate heterogeneity and near 75% or larger reflect high heterogeneity. In the case of significant heterogeneity (*P* < .05 or *I*^2^ > 50%), a random-effects meta-analysis (DerSimonian and Laird method) was adopted to generate the pooled prevalence and 95% confidence intervals, otherwise, a Mantel-Haenszel fixed-effects model was adopted. Leave-one-out sensitivity analysis was conducted to assess the robustness of the pooled results.

#### 2.5.7 Data synthesis

The results of the study will be analyzed by RevMan 5.0 software provided by Cochrane collaborate on network. The binary data will be expressed by the odds ratio, while the continuous data will use the mean difference.

#### 2.5.8 Subgroup analysis

When the heterogeneity test results are heterogeneous, we need to clarify the source of the heterogeneity by subgroup analysis. First, we did univariable meta-regression to test the individual association of prevalence estimates and relevant factors, which included age, sex (male vs female), setting, study year, and geographic indicators (latitude and longitude). Factors that were significantly associated with the prevalence of DED in the univariable meta-regression were subsequently included in the multivariable meta-regression analysis. To visually inspect the goodness of the final multivariable regression, diagnostic plots were generated, including predicted vs standardized residuals, Quantile-Quantile (QQ) plot and histogram of residuals.

#### 2.5.9 Sensitivity analysis

Sensitivity analysis can not only assess the stability and reliability of the conclusions of the meta analysis, but also assess whether the changes in the results are related to the impact of a single study. If the stability of the conclusion is poor, we can achieve the purpose of increasing stability by changing the analysis model, inclusion and exclusion criteria, or excluding a certain type of literature.

### 2.6 Ethics and dissemination

We will publish the system review results in peer-reviewed journals, disseminated in meetings or in peer-reviewed publications. Aggregated published data will be used to exclude data of individuals, so there is no need for obtaining the ethical approval or patients’ informed consent.

## 3 Discussion

Age and female sex have been found to be the greatest risk factors for DED. This is supported by the results of clinical studies of reduced tear secretion in women in the sixth decade.^[21,22]^ With biological aging, the decrease of tear secretion and the increase of evaporation may be the core explanation of DED.^[23,24]^ The function and structure of lacrimal gland (LG) were impaired apparently with aging. The histopathologic changes such as acinar atrophy, periacinar fibrosis, periductal fibrosis can be observed in the human main LG.^[25,26,27,28]^ Diffuse fibrosis and diffuse atrophy in orbital lobes were more frequently observed in women than in men.^[28,29]^ Decrease of tear volume and increase of tear break-up time in age-related eyelid alterations like lid laxity, meibomian gland atrophy, and orifice metaplasia may related to dry eye.^[30–34]^ It has been reported that Marx line, the meibomian gland orifices located anterior to the mucocutaneous junction in young healthy eyelid, migrate anteriorly with aging.^[35]^ Meibomian adenosis can occur alone or associated with tear deficiency (aqueous-tear deficiency).

As life expectancy increases, the immune system also ages and there is increasing evidence that DED is associated with inflammation. Compared with the control group, the levels of inflammatory mediators and T cell related mediators (such as IL-1 β, IL-6, TNF - α, IL-17, IFN - γ) in conjunctiva and tears of patients with DED increased.^[36–38]^ Although previous studies have shown that DED is an age-related disease, there are still controversies about whether aging leads to DED, or whether DED is an aging disease with completely different mechanisms from aging.^[39]^ In a conclusion, it is more and more obvious that, similar to autoimmune DED, age-related DED has significant inflammatory response and complex immune response, and overtime will lead to deep LG and ocular surface changes.

## Author contributions

**Conceptualization:** Xinyue Zhang, Lizhen Wang, Xiaoying Huang, Yanlin Zheng

**Data curation:** Xiaoying Huang, Lu Deng

**Formal analysis:** Xinyue Zhang, Lizhen Wang

**Methodology:** Xinyue Zhang, Lizhen Wang

**Project administration:** Yanlin Zheng

**Resources:** Xinyue Zhang, Lizhen Wang, Xiaoying Huang, Lu Deng

**Software:** Xinyue Zhang, Lizhen Wang

**Supervision:** Yanlin Zheng

**Writing – original draft:** Xinyue Zhang, Lizhen Wang

**Writing – review & editing:** Yanlin Zheng
